# Hypothalamic injury patterns after resection of craniopharyngiomas and correlation to tumor origin: A study based on endoscopic observation

**DOI:** 10.1002/cam4.3589

**Published:** 2020-11-03

**Authors:** Le Yang, ShenHao Xie, Bin Tang, Xiao Wu, ZhiGao Tong, Chao Fang, Han Ding, YouYuan Bao, SuYue Zheng, Tao Hong

**Affiliations:** ^1^ Department of Neurosurgery The First Affiliated Hospital of Nanchang University Nanchang China

**Keywords:** craniopharyngioma, endoscopic endonasal approach, hypothalamic injury, neuropsychological function, surgical outcome, tumor origin

## Abstract

The precise understanding of hypothalamic injury (HI) patterns and their relationship with different craniopharyngioma (CP) classifications remains poorly addressed. Here, four HI patterns after CP resection based on endoscopic observation were introduced. A total of 131 CP cases treated with endoscopic endonasal approach (EEA) were reviewed retrospectively and divided into four HI patterns: no‐HI, mild‐HI, unilateral‐HI and bilateral‐HI, according to intraoperative findings. The outcomes were evaluated and compared between groups in terms of weight gain, endocrine status, electrolyte disturbance and neuropsychological function before and after surgery. A systematic correlation was found between CP origin and subsequent HI patterns. The majority of intrasellar and suprasellar stalk origins lead to a no‐HI pattern, the central‐type CP mainly develops a mild or bilateral HI pattern, and the majority of tumors with hypothalamic stalk origins result in unilateral HI and sometimes bilateral HI patterns. The proportion of tumors with a maximum diameter >3 cm in the no‐HI group was higher than that in the mild‐HI group, BMI and quality of life in the no‐HI group showed better results than those in the other groups. The incidence of new‐onset diabetes insipidus in the bilateral‐HI group was significantly higher than that in the other groups. Memory difficulty was observed mainly in the unilateral‐HI and bilateral‐HI groups. However, the outcomes of electrolyte disturbance, sleep, and cognitive disorder in the unilateral‐HI group were significantly better than those in the bilateral‐HI group. This study suggests the possibility of using pre‐ and intraoperative observation of CP origin to predict four HI patterns and even subsequent outcomes after tumor removal.

## Graphical abstract

This study provided possibility to use the pre‐ and intraoperative observation of craniopharyngiomas origin to predict the four hypothalamic injury patterns and subsequent outcomes after tumor removal.

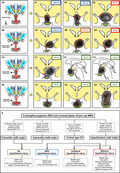

## 1 INTRODUCTION

The hypothalamus is a small multinuclear complex at the brain base. It is the main governing center for homeostatic functions that are of vital importance for survival, such as food and water intake, temperature regulation, sleepiness, memory, cognition, depression, anxiety and control of pituitary function.^1^, ^2^, ^3^ Craniopharyngiomas (CPs), tumors that may originate at different positions along the hypothalamus‐pituitary axis during the embryonic period, are a heterogeneous disease comprising different risk factors for hypothalamic injury (HI). Hypothalamic involvement and surgical injury to hypothalamic structures always lead to sequelae such as impaired physical and social functionality,^4^, ^5^, ^6^ with a major negative impact on quality of life (QoL) in surviving patients.^4^, ^5^, ^6^, ^7^, ^8^ However, the preservation of hypothalamic function varies with different degrees of HI. Data are still scarce in analyzing the differences in outcomes between mild and extensive HI or the compensatory effectiveness of the contralateral‐intact hypothalamus in regard to unilateral HI. Hence, an explicit and visible definition of HI is of crucial importance to the understanding of short‐ and long‐term neurological sequelae and decreases in QoL.^9^, ^10^

For many years, the evaluation of hypothalamic involvement was limited in indirect observation of neuroimaging or CP classifications based on a transcranial approach. However, numerous transcranial classifications categorizing CP into different subtypes have been suggested, mainly based on the topographical relationship between tumors and the optic chiasm or the third ventricle along with the tumor size. Therefore, uncertainties still exist in identifying the exact tumor origin or understanding the precise relationship between the tumor and surrounding structures (the hypothalamus in particular) due to obstruction of structures such as the optic nerve, chiasm, and carotid artery as well as the narrow operative view during transcranial approaches. Previous studies^11^, ^12^, ^13^, ^14^, ^15^ have suggested that radical resection with lower recurrence rates may result in higher risk of morbidity; this dilemma must be balanced against the indications for partial resection to preserve the hypothalamic structures.^16^ The endoscopic endonasal approach (EEA) showed inherent merits, including the inline corridor, superior illumination, and wide‐angled surgical view without blind corners. It has been proposed that the EEA can potentially maintain or slightly improve the QoL simultaneously with high gross total resection (GTR).^17^ Moreover, the origin site of the tumor and hypothalamic involvement (invasion or compression) could be clearly and distinctly visible in CP classification based on the EEA. More importantly, since the angle of tumor invasion to the hypothalamus is coaxial with endoscopy, the surgical view of HI would be fully exposed. In a previous paper^18^ based on preoperative imaging and EEA observation, four typical tumor origins were summarized, including central‐type CP, hypothalamic stalk origin, suprasellar stalk origin and intrasellar stalk origin. Nevertheless, tumors of different origins along the hypothalamic‐pituitary axis always involve different anatomical structures and lead to different HI patterns after tumor removal. Thus, defining the correlation of different CP origins and the subsequent HI patterns may be clinical significance but remains inconclusive.

This work aimed to summarize a series of HI patterns after CP resection based on endoscopic observation. We summarized the relationship between HI patterns and various CP origins and then analyzed the outcomes, including weight gain, endocrine status, diabetes insipidus (DI), postoperative hypernatremia, sleepiness, memory, cognition, depression, anxiety and QoL, with different HI patterns. We hope that our work may serve as a reference for future studies on the prediction of HI patterns and outcomes of CP patients.

## 2 MATERIALS AND METHODS

### 2.1 Participants

A total of 133 CP patients who underwent EEA resection between December 2012 and March 2019 were identified. The postoperative mortality rate, defined as death within 30 days postoperatively, was 1.5% (2/133); one patient with infundibular‐intraventricular partially cystic tumor with dense adhesion to the floor of the third ventricle died after 9 days after operation; the other one patient died due to postoperative pneumonia. All 131 patients who underwent endoscopic endonasal approach for CPs were retrospectively reviewed. The mean follow‐up duration of the entire cohort was 34.3 months (range 9–82 months). Hospital review board approval was obtained, and informed consent was obtained from all patients included in this study. All surgeries were performed by the chief surgeon (T.H.) and assisted by attending surgeons (B.T., S.H.X. and L.Y.).

### 2.2 Surgical procedures

A 2‐surgeon 4‐handed technique with binostril access was employed as previously described.^17^, ^19^ Under strict adherence to the principle of direct visualization, tumor resection was carried out by recognition and separation along the border of the tumor and hypothalamus. Tumor removal was assessed based on the surgeon's intraoperative findings and the results of the postoperative contrast‐enhanced MRI study. Identifying the origin of the tumor was the most crucial step throughout the operation with EEA.

**FIGURE 1:**
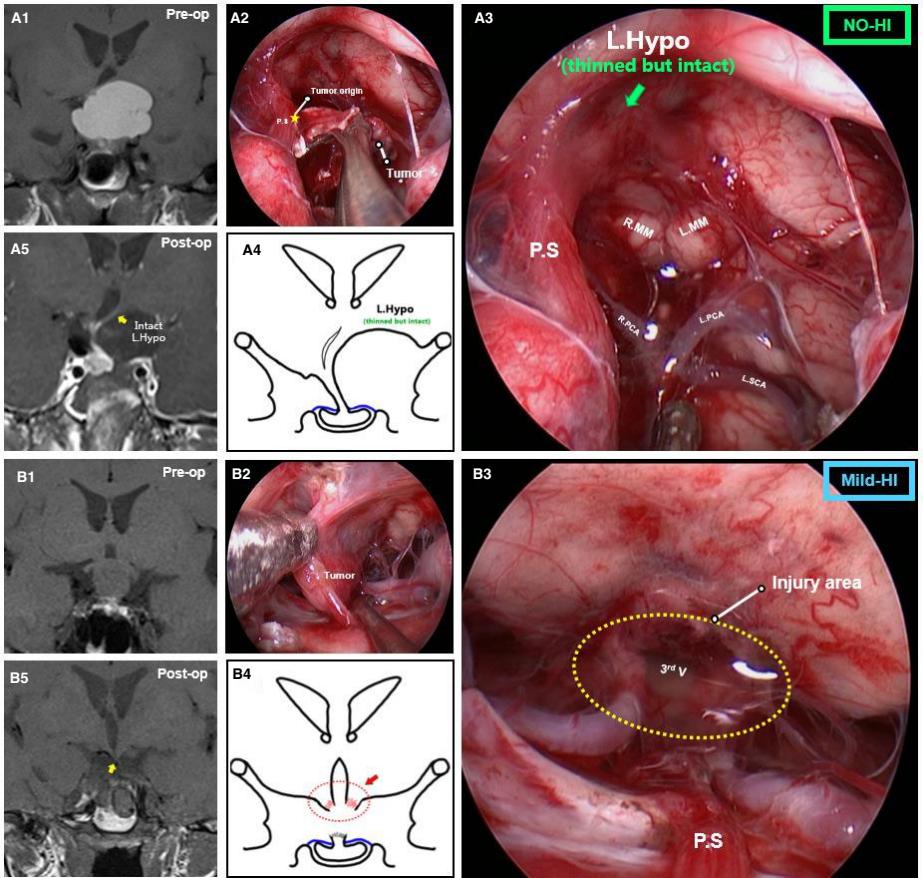
Pre‐ and post‐operative images and intraoperative findings of NO‐HI and Mild‐HI patterns

**FIGURE 2:**
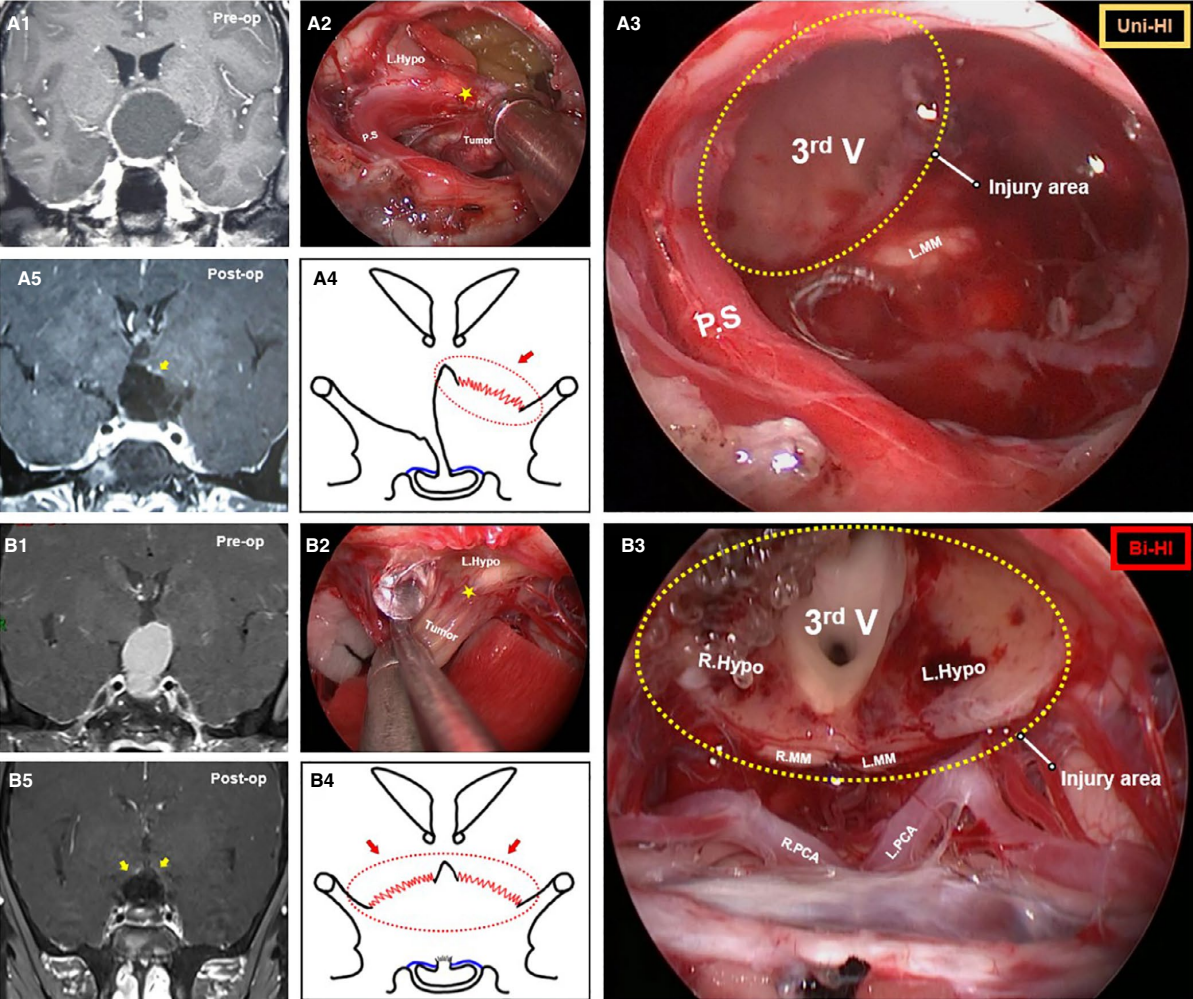
Pre‐ and post‐operative images and intraoperative findings of Unilateral‐HI and Bilateral‐HI patterns

Since the origin of CPs could develop anywhere from the junction of the hypothalamus and the top of the parasellar region (PS) to the intrasellar segment, generally, the tumor would occur either in the form of a central‐type CP or in the form of origin from one of the three areas (junction of the hypothalamus and PS, low portion of the suprasellar stalk or intrasellar stalk) we observed under EEA. To better investigate the correlation between CP origin and HI pattern, the CP origin in each patient was categorized into four types as previously described^18^, ^20^: (a) Central‐type CP: the tumor expanded within and along the stalk, had no pedicle or definite origin site could be identified; (b) Hypothalamic stalk origin: the tumor developed at the junction of the hypothalamus and PS, commonly extended up to hypothalamus and/or down to the upper PS. Sometimes invaded into the third ventricle, corresponding to the “tubero‐infundibular topography” that was proposed previously^21^, ^22^; (c) Suprasellar stalk origin: the tumor derived from suprasellar PS segment (lower part of PS) and in general located extraventricularly; and (d) Intrasellar stalk origin: the tumor originated from the bottom part of the PS under the diaphragma. The exact HI extent was observed and categorized with the EEA after tumor removal in each patient with four patterns: (a) No‐HI: The entire tumor was located extra‐ventricularly, and the intactness of the hypothalamus was preserved postoperatively. In preoperative MRI, the 3^rd^ ventricle and hypothalamus were compressed to one side (Figure 1A1). During the operation, the margin between the hypothalamus and the tumor was clearly identified, and the adhesive tumor could be slowly separated (Figure 1A2). After tumor removal, the compressed surface of the hypothalamus was thinned but still intact. In addition, the intact floor of the 3^rd^ ventricle and mammillary bodies were visible but compressed upward (Figure 1A3), and the position of the compressed hypothalamus recovered postoperatively (Figure 1A4,A5); (b) Mild‐HI: Commonly the tumors were not very large. After tumor removal, the extent of HI and third ventricle opening were limited in the tuberoinfundibular region, and most of the bilateral hypothalamus was preserved (Figure 1B1‐B5); (c) Unilateral HI: The tumor invasion was severe (Figure 2A2), compressing the 3^rd^ ventricle and PS into the contralateral side away from the midline in preoperative MRI (Figure 2A1). After tumor removal, the injury extent was limited to one side of the hypothalamus, and the remnant of PS and the nearly intact contralateral hypothalamus were preserved (Figure 2A3‐A5); (d) Bilateral‐HI: The majority of the invasion cases occurred in this category. The tumor always grew in a vertical pattern and had a larger volume in the preoperative MRI (Figure 2B1), the invasion always extended into almost the entire hypothalamus (Figure 2B2), and nearly no hypothalamus was preserved after GTR. The third ventricle was widely open, and the interthalamic adhesion was visible (Figure 2B3‐B5). The videos were assessed by surgeons (X.W., Z.G.T. and C.F.) who were blinded to the clinical outcome and blinded to each other.

### 2.3 Measurement and neuropsychological function

Before surgery, the maximum tumor diameter in each patient was determined by preoperative MRI. Endocrinological evaluation was performed before and after surgery as described previously.^18^ Partial hypopituitarism was defined as hormone deficiencies in one or two axes, and panhypopituitarism was defined as hormone deficiencies in three or more axes. Diabetes insipidus (DI) was diagnosed before and after surgery in patients who had polydipsia and polyuria with urine‐specific gravity <1.005 and urine osmolality <300 mOsm/kg, as well as a positive response to desmopressin. The percentage of new anterior pituitary dysfunction was calculated as the proportion of postoperative new‐onset patients to preoperative normal endocrine level patients. New DI was calculated based on the patients without DI preoperatively, and the deterioration of pituitary dysfunction was calculated based on the preoperative partial hypopituitarism group who developed with new deficiencies in other axes. BMI was calculated with the formula for the adult patients: BMI = [kg weight]/[height in meters]^2^. The BMI z‐scores or BMI standard deviation scores (SDS) were considered in the patients with childhood onset (<18 year old) to calculate the relative weight adjustment according to the child's age and sex.^23^

Neuropsychological function was evaluated pre‐ and postoperatively as follows: subjective daytime sleepiness was assessed with the Epworth Sleepiness Scale (ESS),^24^, ^25^ Everyday Memory Questionnaire‐Revised (EMQ‐R)^26^ was used to evaluate self‐reported memory difficulties, cognitive function was evaluated using the Montreal Cognitive Assessment (MOCA),^27^ depression and anxiety were assessed by the Hamilton Depression Rating Scales (HDRS)^28^ and Hamilton Anxiety Rating Scales (HARS),^29^ and QoL was assessed with the anterior skull base quality‐of‐life questionnaire (ASBQ),^30^ which consists of 35 questions and higher scores indicate a better QoL. Data were presented with ∆ values of different scales (∆ = the difference of values between postop and preop).

### 2.4 Statistical analysis

Differences between groups were assessed by the Chi‐square test for categorical variables with Bonferroni correction, and one‐way ANOVA was used to compare continuous variables followed by LSD multiple comparisons. The data are presented as the mean±SEM. Statistical analysis of the data was performed using IBM SPSS Statistics version 21. The results were considered significant at *p* < 0.05.

## 3 RESULT

### 3.1 Patient characteristics

Among all 131 patients (74 men and 57 women, range 9–70 years), 19 had previously been surgically treated before admission. Of these 19 patients, 14 had previously undergone surgery via a transcranial approach, followed by gamma knife therapy in five patients. Among them, four patients had undergone transsphenoidal microsurgery, and another patient had previously undergone gamma knife surgery and stereotactic cyst aspiration at another institution. The most common clinical manifestation was pituitary dysfunction, which occurred in 85 patients (64.9%), followed by visual impairment (62.6%), headache (61.8%), and DI (24.4%).

### 3.2 Correlation between HI patterns and tumor origins

**FIGURE 3:**
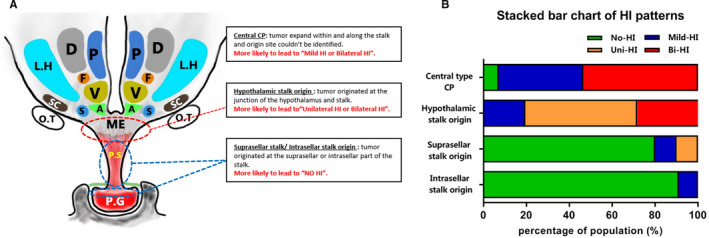
Scheme of different CP origin and the correlation between different CP origins and HI patterns

Of the 131 patients in the present series, 39 (29.8%) were categorized as no‐HI pattern, 30 (22.9%) as mild‐HI pattern, 26 (19.8%) as unilateral‐HI pattern and 36 (27.5%) as bilateral‐HI pattern. When the tumor was classified as central‐type CP, no specific origins could be identified, and the tumor extended through the center of the PS. Among our 43 central‐type CP patients, 17 (39.5%) had mild HI, and 23 (54.5%) had bilateral HI. Only three (6%) patients were classified as no‐HI due to their small tumor volume. All the patients (46 in total) with hypothalamic stalk origin had HI, and the majority of the cohort had unilateral‐HI and bilateral‐HI patterns (52.2% and 28.3%, respectively). In patients with suprasellar stalk origin and intrasellar stalk origin, by virtue of the origins located in the middle and bottom of the PS, the hypothalamus remained intact in most of these cases, which were categorized into the no‐HI group (Table 1, Figure 3A,B).

**TABLE 1:** Correlation between HI patterns and tumor origins

	Central type (n = 43)	Hypothalamic stalk origin (n = 46)	Suprasellar stalk origin (n = 20)	Intrasellar stalk origin (n = 22)
NO‐HI, n (%)	3 (7.0)	0 (0)	16 (80)	20 (90.9)
Mild‐HI, n (%)	17 (25.6)	9 (19.6)	2 (10)	2 (9.1)
Unilateral‐HI, n (%)	0 (0)	24 (52.1)	2 (10)	0 (0)
Bilateral‐HI, n (%)	23 (53.4)	13 (28.3)	0 (0)	0 (0)

When the *p* value is very small and less than 0.01, we describe it as "*p*＜0.01" (in bold).

### 3.3 Tumor volume, weight gain, and endocrine and neuropsychological outcomes

In the no‐HI group, 66.7% of patients had a maximum tumor diameter >3 cm. However, the mild‐HI group with subtle hypothalamic lesions accounted for only 16.7% compared with no‐HI group (*p* < 0.05). This result indicated that the tumor size and incidence of HI were not always correlated (Table 2). In our series, adamantinomatous CP (ACP) is more common than papillary CP (PCP) in all HI groups. Among all patients, PS preservation rate was significant lower in bilateral‐HI group (27.8%) when compared with no‐HI group (*p* < 0.05). New anterior pituitary dysfunction was observed in the majority of patients with normal pituitary function preoperatively in each group, but for deterioration of pituitary dysfunction, the unilateral‐HI and bilateral‐HI group showed higher incidence compared with no‐HI group (*p* < 0.05). Regarding postoperative weight gain, the increase in BMI in the no‐HI group was 4.44 ± 3.00%, which was significantly less than that in the other three groups with HI (*p* < 0.05, Table 3). In the QoL assessment, we observed that in the ASBQ questionnaire, the no‐HI group showed better outcomes than the other groups (*p* < 0.05, Table 3). However, in postoperative hypernatremia, sleepiness and cognition evaluations, the bilateral‐HI group performed worse than any of the other three groups (Tables 2, 3). As to the memory questionnaire, both the unilateral‐HI and bilateral‐HI groups were significantly worse than the no‐HI and mild‐HI groups. In the aspects of anxiety and depression, there was no significant difference between groups (Table 3).

**TABLE 2:** Characteristics and endocrine outcomes of recruited CP patients graded in four HI patterns

	NO‐HI (n = 39)	Mild‐HI (n = 30)	Unilateral‐HI (n = 26)	Bilateral‐HI (n = 36)	*p* value
Age (Adults/Children)	25/14	24/6	21/5	29/7	0.266
Gender (female/male)	11/28	16/14	12/14	18/18	0.134
Recurrence rate, n (%)	1(2.6)	0(0)	2(7.7)	0(0)	0.175
Tumor maximum diameter>3 cm, n (%)	26(66.7)	**5(16.7)** *	20(76.9)	30(83.3)	**<0.01**
Gross total resection, n (%)	36(92.3)	28(93.3)	23(88.5)	32(88.9)	0.925
Histological type (ACP/PCP)	35/4	27/3	25/1	34/2	0.713
PS preservation, n (%)	31(79.5)	18(60)	20(76.9)	**10(27.8)** *	**<0.01**
Hydrocephalus, n (%)	2(5.1)	0(0)	4(15.4)	2(5.6)	0.115
Post‐op CSF leak, n (%)	0(0)	1(3.3)	3(11.5)	3(8.3)	0.168
Post‐op epilepsy, n (%)	0(0)	0(0)	3(11.5)	2(5.6)	0.064
New anterior pituitary dysfunction, n (%)^①^	17(73.9)	14(70)	9(81.8)	5(100)	0.528
Deterioration of pituitary dysfunction, n (%)^②^	3(20)	2(33.3)	**11(84.6)** *	**5(62.5)** *	**<0.01**
New‐onset DI, n (%)^③^	20(71.4)	20(80)	17(85)	25(96.2)	0.111
Post‐op Hypernatremia, n (%)	7(17.9)	5(16.7)	5(19.2)	**20(55.6)** *	**<0.01**
Post‐op Hyponatremia, n (%)	0(0)	3(10)	2(7.7)	2(5.6)	0.287

ACP, adamantinomatous craniopharyngioma; CSF, cerebrospinal fluid; DI, diabetes insipidus; PCP, papillary craniopharyngioma; PS, pituitary stalk.

①: % means “% of Pre‐op normal.” ②: % means “% of Preop‐Partial Hypo.” ③: % means “% of Pre‐op normal.

* *p* < 0.05 vs. NO‐HI group.

**TABLE 3:** Weight gain and neuropsychological function of post‐op CP patients in different HI patterns

	NO HI	Mild HI	Unilateral HI	Bilateral HI	*p* value
Gain of BMI (%)	4.44 ± 3.00	**7.01 ± 4.63** *	**6.73 ± 3.99** *	**7.28 ± 4.29** *	**<0.01**
∆HARS	2.23 ± 1.29	1.83 ± 1.37	2.12 ± 0.95	2.44 ± 1.42	<0.287
∆HDRS	2.21 ± 1.79	1.67 ± 1.32	2.00 ± 1.13	2.19 ± 1.63	0.461
∆ESS	1.64 ± 1.22	2.77 ± 1.52	3.31 ± 1.38	**4.03 ± 2.34** *	**<0.01**
∆MoCA	0.77 ± 0.87	0.52 ± 0.29	0.45 ± 0.32	**1.78 ± 1.69** *	**<0.01**
∆ASBQ	0.21 ± 0.24	**0.52 ± 0.29** *	**0.45 ± 0.32** *	**0.54 ± 0.33** *	**<0.01**
∆EMQ‐R	2.51 ± 2.02	2.73 ± 2.03	**6.92 ± 3.16** *	**6.83 ± 2.70** *	**<0.01**

When the *p* value is very small and less than 0.01, we describe it as "*p*＜0.01" (in bold).

* *p* < 0.05 vs. NO‐HI group.

## 4 DISCUSSION

Various CP classifications^31^, ^32^, ^33^, ^34^, ^35^, ^36^, ^37^ have been proposed for planning the transcranial approach based on CP size and topography relative to the optic chiasm, sellar diaphragm or tumor involvement of the third ventricle floor and third ventricle cavity. However, most of these schemes did not provide any accurate information about the HI patterns after tumor removal. With respect to a prior HI grading system based on brain imaging, Puget et al.^38^ evaluated the degree of hypothalamic involvement postoperatively as follows: (a) no damage; (b) negligible damage; and (c) significant damage according to the identifiability of the third ventricle. Bogusz et al.^39^ evaluated the HI preoperatively as (a) no involvement, (b) anterior involvement and (c) anterior +posterior involvement by taking the mammillary bodies as the boundary of the posterior hypothalamus. In addition, Roth et al.^40^, ^41^ developed a novel hypothalamic lesion scoring system by assessing the lesion extent and relevant nuclei to predict the risk for the development of hypothalamic obesity. Moreover, Pascual et al.^37^ introduced the mammillary body angle (MBA), which is a useful sign for preoperatively differentiating a primary intraventricular CP originating at the infundibulotuberal area from a primary suprasellar CP developed below the third ventricle floor. However, these indirect imaging schemes lack objectivity, assessment results often vary among observers, and the exact extent of HI remains uncertain. However, here, the hypothalamic injury patterns by direct intra‐operative observation was proposed for the first time. The EEA provided a definite vision of the hypothalamic structure with superior illumination, which allowed us to see and grade the exact HI extending into patterns from the surgical field. The CP origin can not only be recognized from a coaxial angle to the tumor invasion, but the EEA can also make it possible to correlate the CP origin and the subsequent HI pattern in each patient: (a) the central‐type CP always leads to bilateral HI, and the degree depends on mild or extensive kind; (b) the suprasellar or intrasellar stalk origin of CP mostly preserved the intact hypothalamus; and (c) the hypothalamic stalk origin of CP can lead to various HI patterns, but the majority lead to unilateral HI and bilateral HI.

### 4.1 Origins and HI patterns

**FIGURE 4:**
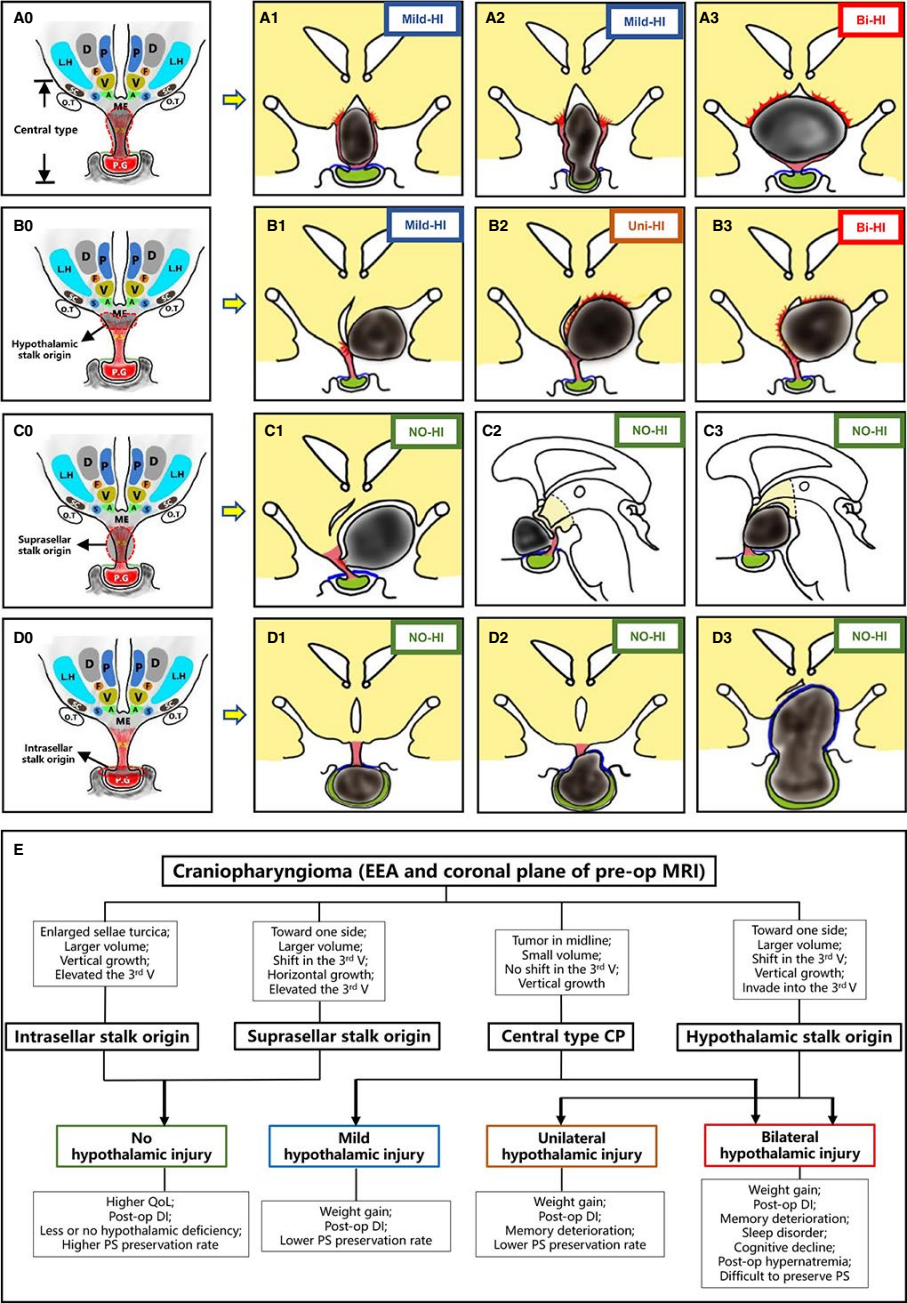
Illustration of different growth patterns of CP origin classification and the HI patterns that might lead to

Here, we summarized four CP origins. However, the growth patterns vary in each origin subtype. With central type CP (Figure 4A0), the tumor might invade the ventral hypothalamus to different degrees (Figure 4A1,A3) and always leads to mild‐HI or bilateral‐HI, sometimes extending downward into the intrasellar region and compressing the pituitary gland (Figure 4A2). For tumors of hypothalamic stalk origin (Figure 4B0), the HI patterns vary in different situations. Sometimes the whole tumor develops with a narrow pedicle, lead to mild‐HI pattern and resulting in mild‐HI after resection (Figure 4B1). However, when the tumor origin was confined to one side of the infundibulum‐tuber cinereum, the tumor invaded the ipsilateral part of the hypothalamus and always led to a unilateral‐HI pattern (Figure 4B2). In addition, in some cases, the origin was located in the middle part of the infundibulum‐tuber cinereum, and the tumor invaded both sides of the hypothalamus, which might occur with bilateral HI after GTR (Figure 4B3). For CPs originating from the lower portion of the suprasellar stalk (Figure 4C0), since the lower origin site led to the infundibulum‐tuber cinereum area, the tumors always developed in the extraventricular space. The tumor expanded from the stalk and pushed the surrounding structures of the PS, including the floor of the hypothalamus and the optic chiasma, always compressing the hypothalamus to one side (Figure 4C1) or led to pre‐ or retroinfundibular types (Figure 4C2, C3), and the hypothalamus remained intact after resection. When the origin spot was located in the subdiaphragmatic portion of the PS (Figure 4D0), which is frequently seen in children, the tumor could extend to the suprasellar space, cavernous sinus and sphenoid sinus, and it has been suspected that the direction of growth depends on the thickness of the sellar diaphragma, inner wall of the cavernous sinus and floor of the sellar turcica. The sellae turcica was always enlarged (Figure 4 D1) and sometimes even grew vertically and pushed up the diaphragma and the hypothalamus with a large volume (Figure 4D3). However, the boundary of the tumor was still confined under the diaphragma, and the hypothalamus was just deformed but would still remain intact postoperatively in these cases. In addition, the subdiaphragma CP might grow along the stalk and break through the foramen of the diaphragma sellar turcica into the suprasellar space (Figure 4D2). In these cases, merely removing the main part of CP in the subdiaphragmatic space is insufficient, and the suprasellar compartment must be check and clearance excision of the remnant tumor is absolutely necessary to prevent recurrence. Here, our present study suggested the possibility of using pre‐ and intraoperative observations of CP origin to predict HI patterns and even subsequent outcomes after GTR. In other words, by speculating from the preoperative MRI and confirming the operation under EEA, the tumor origin could be clearly identified. Intrasellar and suprasellar stalk origins always lead to the no‐HI pattern, central‐type CP always develops with mild or bilateral HI, and the majority of tumors with hypothalamic stalk origins result in unilateral HI and sometimes bilateral HI patterns (see Figure 4E). Thus, it is of great beneficial effect for the prediction of clinical outcomes and serves as an important reference for treatment.

### 4.2 HI patterns and outcomes

In previous literature, the CP size was hypothesized to be associated with risks of operation and deceased outcome.^42^ However, here, the no‐HI group exhibited a larger tumor size than the mild‐HI group, which had a worse outcome. It revealed that as long as the CP origin was from the lower portion of the PS, the outcome of patients might be better even if the tumor size was large. The tumor size and prognosis were not always correlated, which suggests that the recognition of tumor origin but not tumor size is the most critical factor. The PCPs are much rarer than ACPs and are pathologically distinct,^43^ PCPs are rarely calcified, mostly solid, better circumscribed and usually considered to be less aggressive than ACPs.^44^ In our data, majority (92.4%) of the cases are ACPs, but only 10 PCPs were observed in all groups (Table 2). Thus, more PCPs are needed to analysis the relationship between the two histopathological variants and HI patterns in future. Few studies^45^, ^46^ evaluated the influence of CP involving the third ventricle and a tight tumor adherence to surrounding neurovascular structures were more likely lead to recurrence, whereas in our data HI patterns did not proved to be a determinant of CP recurrence. We believe that safely achieving a higher GTR will be a good option to avoid recurrence even with relative more severe hypothalamic involvement. Regarding QoL, only the no‐HI group showed a better result in our data, which further indicated that to a larger extent, the exact origin of CP determines the postoperative outcome instead of the tumor size. Hypothalamic obesity has profound negative consequences for both psychosocial function and metabolic abnormalities.^47^, ^48^ The generally accepted cause of hypothalamic obesity in patients with CP is damage to the ventromedial hypothalamus, which is responsible for appetite regulation and fat composition.^49^ Here, the mild‐HI, unilateral‐HI and bilateral‐HI groups showed significant increases compared to the no‐HI group, suggesting that HI causes obesity independent of different extents.

### 4.3 Resection extent and hypothalamus

GTR rate must be balanced against recurrence and mortality after surgery. Here in our data with EEA, the mortality was 1.5%. In another research by Guo et al.^50^ with 335 CP patients, the surgical mortality rate was 2.69% with transcranial approach but only with less than 80% of GTR rate. So precise operating skills to minimize brain damage and close perioperative nursing is very important to reduce mortality. Besides, GTR rate is an important factor related to recurrence and post‐op HI risks. We achieved GTR rate of 90.8% in present study, but when dealing the cases with unacceptable risk of HI, for instance, in a few cases the tumor with huge, dense and very thin capsule expanded widely to other structures, or sometimes the tumor infiltrated and replaced extensively into most of the hypothalamus and 3 V, incomplete resections were adopted for the sake of post‐op quality of life. When dealing with the incomplete resection cases, radiotherapy and postoperative MRI check are the main plan. Recently, Prieto et al. proposed a comprehensive topographical categorization of CP adherence that can be used to anticipate the surgical risk of hypothalamic injury and to plan the degree of removal.^51^ Here, if the tumor origin spot located outside the 3^rd^ ventricle, most of the CP adherence to the surrounding structures is “sessile” or “cap‐like” patterns which usually have a clear cleavage plane. Thus, blunt separation is recommended to minimize the damage and might accompanied with a better outcome with less hypothalamic obesity and neurological dysfunction. However, if the central type CP that originated inside the stalk and invaded into 3^rd^ ventricle or diaphragma sellae, it might lead to “ring‐like” or “circumferential” adherences, sharp dissection along the border of tumor and hypothalamus was recommended. In these cases, 3rd ventricle stayed open and sometimes PS was sacrificed, always lead to bilateral HI and relative worse outcome.

### 4.4 Hypothalamic nucleus in CP invasion

The infundibularis nucleus, which correspond to hypothalamic arcuate nucleus in animal, is located in the ventral border of the hypothalamus in close proximity to the median eminence and its energy‐balance circuit. It plays a key role in regulating body weight as it is one of the most important sites in the hypothalamic integration of energy balance and innervating the second‐order neuronal signaling includes ventromedial nucleus, paraventricular nucleus and perifornical area.^52^ So, it is believed that the involvement of the infundibularis nucleus in HI (even to a mild extent) might lead to obesity. The present data showed that new‐onset DI occurrence was the highest in the bilateral‐HI group. Since the neuroendocrine system also depends on the PS and pituitary, in many cases, the preservation of PS is not promising, and the anterior pituitary is always compressed and affected in intrasellar‐origin CP, so neuroendocrine function should be assessed with the whole hypothalamus‐pituitary axis. Here, the proportion of postoperative hypernatremia was significantly higher in the bilateral‐HI group than in the other groups. Moreover, the evaluation of sleep disorders and cognition were worse only in the bilateral‐HI group. It may be speculated that a greater impact would occur on electrolyte disturbance, sleepiness and cognition in extensive bilateral HI compared with bilateral HI. Considering the preservation of the above‐mentioned function, the intact‐remnant contralateral hypothalamus may compensate for the defect of the injured hypothalamus to a certain extent. Therefore, protection of the intactness of the contralateral hypothalamus may serve as a new solution in functional preservation. The hypothalamus is a collection of multiple specialized nuclei, integrating signals from the viscera, bloodstream, brainstem, and retina, and subsequently exerts control over the hemostasis of the body via hormonal and autonomic output.^53^ Disruption of these signals may cause sleep disorders and somnolence associated with either melatonin deficiency or disruption of normal circadian rhythm. However, in the memory investigation, the mammillary bodies always affected due to the injury extent in the unilateral and bilateral HI pattern. Our results showed that both the unilateral‐HI and bilateral‐HI groups were worse than the no‐HI and mild‐HI groups, which is similar with the results from Pasucal et al.^54^ that severe memory deficits occurred with damage of the mammillary bodies. Nevertheless, the functional impairment following disturbance of hypothalamic tracts is also an important topic in exploring the hypothalamic injury, and further work is required to investigate the connecting tract between different brain regions and changes in functional parameters with DTI and fMRI studies. Because the fornix and mammary body in the hypothalamus are both important components of the Papez circuit responsible for memory and emotion, and a previous study demonstrated^55^ that smaller hippocampal volume and microstructural white matter alterations in the hippocampus were associated with a decline in general knowledge and episodic visual memory in patients with CP, we speculate that the integrity of the Papez circuit is crucial in memory function and that even unilateral HI can also induce severe memory loss; But one of the limitations in present study is that only the observation of hypothalamus was emphasized. The efferent pathways from hypothalamus and connectivity of different brain regions, for example, the white matter connection of pons‐to‐hypothalamic tract, cerebello‐hypothalamic pathway or Papez circuit were not further analyzed here. Thus, deeper investigation refers to the changes in those microstructures is needed in the future.

## 5 CONCLUSION

Here, we proposed new insight on HI patterns after resection of CP based on endoscopic observation, including four main categories: no HI, mild HI, unilateral HI and bilateral HI. A systematic correlation was found between CP origin and the subsequent HI patterns, which greatly helped us with operative strategy generation and postoperative management. Additionally, clear recognition of the CP origin rather than the tumor size to a larger extent determines the HI patterns and postoperative QoL. Protecting the intactness of the remnant contralateral hypothalamus is crucial in maintaining electrolyte balance, sleepiness and cognition function due to the compensation effect. Hence, our HI scheme based on the EEA may serve as a new insight into the preservation of hypothalamic function and treatment in patients.

## DISCLOSURE STATEMENT

The authors declare that they have no conflicts interests.

## AUTHOR CONTRIBUTIONS

Conception and design: T.H.; Acquisition of data: L.Y., S.H.X., and B.T.; Analysis and interpretation of data: X.W., C.F., and Z.G.T.; Drafting the article: L.Y.; Critical revising the article: T.H. and L.Y.; Statistical analysis: H.D. and Y.Y.B.; Administrative / technical / material support: S.Y.Z.

## Data Availability

All of the individual participant data included in this study are available upon request by contact with the corresponding author.
